# Skill acquisition as a function of age, hand and task difficulty: Interactions between cognition and action

**DOI:** 10.1371/journal.pone.0211706

**Published:** 2019-02-07

**Authors:** Rachael K. Raw, Richard M. Wilkie, Richard J. Allen, Matthew Warburton, Matteo Leonetti, Justin H. G. Williams, Mark Mon-Williams

**Affiliations:** 1 School of Psychology, University of Leeds, Leeds, United Kingdom; 2 School of Computing, University of Leeds, Leeds, United Kingdom; 3 University of Aberdeen Medical School, Institute of Medical Sciences, Aberdeen, Scotland, United Kingdom; 4 Bradford Institute for Health Research, Bradford, United Kingdom; 5 National Centre for Vision, University of Southeast Norway, Kongsberg, Norway; Curtin University, AUSTRALIA

## Abstract

Some activities can be meaningfully dichotomised as ‘cognitive’ or ‘sensorimotor’ in nature—but many cannot. This has radical implications for understanding activity limitation in disability. For example, older adults take longer to learn the serial order of a complex sequence but also exhibit slower, more variable and inaccurate motor performance. So is their impaired skill acquisition a cognitive or motor deficit? We modelled sequence learning as a process involving a limited capacity buffer (working memory), where reduced performance restricts the number of elements that can be stored. To test this model, we examined the relationship between motor performance and sequence learning. Experiment 1 established that older adults were worse at learning the serial order of a complex sequence. Experiment 2 found that participants showed impaired sequence learning when the non-preferred hand was used. Experiment 3 confirmed that serial order learning is impaired when motor demands increase (as the model predicted). These results can be captured by reinforcement learning frameworks which suggest sequence learning will be constrained both by an individual’s sensorimotor ability and cognitive capacity.

## Introduction

The study of cognition lies at the centre of psychological research. Nevertheless, there is no ‘standard model’ of cognition, and psychology lacks consensus on the very nature of this broad construct. Indeed, there are diametrically opposing views on how we should understand cognition. On the one hand, there is a long tradition of conceptualising ‘cognition’ as a closed system that is concerned purely with abstract information processing; a system which is independent of perceptual-motor functions. This entrenched view of the inconsequential nature of motor control within studies of cognition is particularly evident in the most popular approaches used to administer computerised cognitive test batteries (e.g. the CANTAB, NEPSY-II, NIH Toolbox). These batteries either: (i) ignore the motor aspects of performance once a baseline motor task has been ‘passed’ or (ii) treat motor performance as a separate entity that can be dissociated from other cognitive functions. The CANTAB [1] adopts approach (i) and assays visuomotor control in an initial ‘induction’ test before the core battery of tasks is presented. The core tests are designed to assess specific ‘cognitive’ abilities (e.g. attention, working memory and decision making) but necessarily require a variety of task-specific manual responses. This approach is problematic because it assumes that an individual’s sensorimotor performance does not contribute to variability in any of the subsequent, explicitly ‘cognitive’, assessments (provided motor performance exceeds a minimum threshold on one specific ‘motor’ task at the beginning of the assessment). The alternative approach taken in other test batteries (e.g. the NEPSY-II [2] and the NIH Toolbox [3]) is to treat motor skill as a separate function that can be measured independently. Thus, the NEPSY-II and the NIH Toolboxincorporate comprehensive sub-tests explicitly intended to assess various aspects of motor function. However, they indicate that these are taxonomically distinct from cognitive sub-tests (e.g. the NIH Toolbox includes a sub-battery of ‘Cognitive’ tests and a separate sub-battery of ‘Motor’ tests), and this gives rise to ontological questions with no clear answers–what defines a specific sub-test with ‘cognitive’ but no ‘motoric’ demands, or vice versa?

The separation of ‘cognition’ from the ‘sensorimotor’ system is somewhat formalised in the dual-task literature where the extent to which these purportedly separate systems interfere with each other is explored by asking participants to conduct a cognitive exercise concurrently with a motor task. In contrast there are increasing numbers of researchers who argue that cognition is an open system that can only be understood in terms of its embodiment within the anatomical structures that influence and are influenced by the world [4]. The extreme version of this ‘embodied cognition’ viewpoint posits that cognition is distributed across mind, body and environment and that “concepts, internally represented competence, and knowledge” should be removed as topics of study within cognitive psychology [5].

Theorists have shown that the extreme versions of embodied cognition (i.e. where the environment is viewed as part of the cognitive system) are deeply problematic [4]. Nevertheless, there is a growing consensus that “the mind must be understood in the context of its relationship to a physical body that interacts with the world” [4]. Thus, there is broad support for the notion that it is both useful and appropriate to consider sensorimotor processes as being more than simple mechanisms of output. We suggest that there is a sensible and–importantly–useful middle ground that exists between the idea that cognition is encapsulated from sensorimotor function and the more extreme versions of ‘embodied cognition’. We will use the term ‘Cognition Action Interaction Theory’ to refer to this conceptual meeting ground. Cognition Action Interaction Theory (CAIT) captures the idea that ‘higher-order cognition’: (i) has the sensorimotor system at its phylogenetic and ontogenetic foundations; (ii) is an emergent property of a brain originally constructed for sensorimotor processing; (iii) can operate without the ‘sensorimotor control system’ (and vice versa); (iv) interacts with the ‘sensorimotor system’ in ontogeny and, throughout life, in numerous everyday activities. Thus, CAIT recognises cognition and sensorimotor processing as somewhat separate evolutionary entities (worthy of study in their own right) whilst acknowledging that they are synergistically interdependent.

One implication of this viewpoint is that many complex human activities cannot be meaningfully reduced to their component cognitive and motor parts. We would suggest that it can be meaningful to study cognition in the context of tasks that have minimal motor input (e.g. abstract reasoning about the potential outcomes of a chess move) and also to examine sensorimotor control in the context of tasks that have minimal cognitive input (e.g. picking a fallen chess piece off the floor). Even so, a full understanding of *complex* skilled behaviours in humans (e.g. playing a game of chess) requires a consideration of both the motor and cognitive systems, and how these systems interact. In other words, the impressive repertoire of skills possessed by human adults is not just a testament to the extraordinary neurophysiological organisation that underpins the sensorimotor system. It is the more recently evolved higher-order cognitive architecture that allows Homo Sapiens to display skills that go far beyond the quantity and diversity of skills observed in any other animal species. Thus, the acquisition and production of complex skills in humans rests upon the motor *and* cognitive apparatus working together in unison [6]. This fact is particularly important to recognise when understanding and supporting individuals who show deficits in learning skilled behaviours, because improved understanding of the interaction between motor and cognitive processes can allow for the development of more tailored treatment regimens within a rehabilitation context.

The focus of the present paper is on older adults who often show impaired skill acquisition relative to their younger counterparts. An extensive review of the literature on learning in older adults concluded that complex tasks have a greater likelihood of revealing age differences [7]. Age-related decline in cognitive ability might provide one explanation as to why older adults have particular difficulties with the acquisition of novel skills [8, 9]. This is because the learning of a new skill is likely to require storage and/or attentional resources associated with working memory [10–13]. It is well established that older age is associated with a decline in such cognitive functions (e.g. cognitive slowing, poorer working memory and reduced attention [14, 15]). Moreover, the literature suggests that there are age differences in *how* new skills are acquired. One specific difference is the way in which serial order information is encoded. Studies show that younger adults store parts of a sequence in ‘chunks’, which are internal representations of groups of elements that feature in a given sequence. Encoding sequences in this way saves limited processing resources–instead of recalling each element of a sequence individually, integrated sections of the array (typically three-to-five elements) can be combined and recalled together [16–23]. Older adults do not always benefit from a chunking encoding strategy and show limited chunking compared to younger adults. Even when chunking is used, the chunks are comprised of fewer elements [23, 24]. There is evidence that older adults have generalised difficulties in memorising chunks in tasks involving immediate serial recall [25] and long-term association formation [26–30]. Bo et al. [17] found that older adults had reduced visual working memory capacity and produced shorter chunk lengths in a movement sequence learning task. Positive correlations between working memory and chunk length and between chunk length and sequence learning were also observed (though no direct relationship between working memory and learning rate in older adults was found.).

The decline of working memory in older adults certainly provides a plausible explanation for the problems encountered by older people when faced with the task of learning a new skill. But this does not rule out other factors contributing to a reduction in learning ability. In the past, studies have consistently reported an age-related decline in motor performance, whereby older adults exhibit reduced motor speed and accuracy across a range of manual control and coordination tasks [31–38]. This can be explained by age-related physiological factors such as limited joint flexibility and reduced muscle strength in the limbs [39, 40], neural changes [41–44], increased susceptibility to diseases that affect movement (e.g. stroke, arthritis) and compensatory changes in motor strategy [35, 45]. Little is known about how this decline in underlying motor performance can impact on learning a novel skill. While motor learning undoubtedly relies on the higher-order processes of the cognitive system (i.e. the reasoning and memory processes that allow a new skill to be retained and retrieved [46, 47]), the CAIT perspective would suggest that the motor processes that underlie a particular skill (i.e. the functions of the motor system that allow one to physically move and coordinate one’s fingers to type out a memorised password, for example) must also be considered as an integral part of the learning process.

The aim of the present series of experiments is to better understand the relationship between motor performance and the learning of a skill that requires ‘visual serial order learning’. We capture the features of a complex skill by considering a movement sequence containing a series of sub-goals that need to be achieved in a specific order to produce a given outcome (i.e. the goal). In classic motor learning theory, a central component of learning a complex skill is the ‘cognitive phase’, which entails memorising the requisite movement sequence [48]. Following the cognitive phase is the ‘associative phase’, which involves linking the ‘component parts’ into one smooth perceptual-motor action [48]. Hence, learning a complex skill requires the memorisation of a series of individual movements (i.e. lower-order elements) so that these components can be linked into a smooth action and ultimately become an automated, single, higher-order behaviour [49]. Incidentally there may well be difficulties in defining the lower-order elements for many complex movements; but this issue goes beyond the scope of this manuscript. The modelling of motor learning has been greatly refined over the last four decades, but most motor theorists accept the basic insights provided by Fitts and Posner [48] regarding the key stages involved in movement learning. Indeed, neuroimaging methods have revealed that the release of cognitive control hubs in frontal cortex and cingulate cortex can predict individual differences in rate of learning [50].

The CAIT perspective suggests that motor performance is likely to impact on the memory processes responsible for learning the serial order of the lower-order elements within the cognitive phase. We therefore hypothesised that sequence learning would deteriorate as the elements increased in motor difficulty. It is widely accepted that task difficulty is well indexed by movement duration, so that as an individual’s skill level increases so movement duration decreases. One major reason for decreased movement duration at higher skill levels is because there is less need to make online error corrections. Therefore, movements can be faster when the demands on error correcting mechanisms are reduced (i.e. bandwidth availability decreases when more error corrections are required and vice versa). The lawful nature of this speed-accuracy relationship was first formalised by Fitts [51], though empirical findings led to ‘Fitts’ law’ being reformulated by Welford [52]. There is still some controversy over the precise nature of the function that relates movement duration (time) to task parameters, but Welford’s formulation captures most of the variance in a wide range of experiments (see [53] for a discussion). Welford’s formulation is given in Eq 1 below:

$$MT=a+b\;{Log}_{2}A+c\;{Log}_{2}\;W$$ (1)

Where MT is movement time; A is the amplitude of the movement; W is the size of the target; and a, b and c are constants for a particular individual performing a particular task. It is apparent that increasing the need for error corrections by decreasing the size of the target (W), results in increased movement time (MT). Furthermore, raising the values of a, b and c captures the increase in movement time associated with motor performance decline (i.e. because of increased task difficulty or decreased skill level).

Whilst Eq 1 captures most of the variance associated with a single movement from one target to another, a storage buffer with properties akin to that of working memory is required to model serial learning processes. It has been established that working memory has a limited capacity [13], thus it follows that increasing the level of difficulty of the lower-order elements in a sequence learning task may well decrease the total number of elements that can be held in working memory (i.e. because increased difficulty increases the informational load associated with each element). Accordingly, we can model sequence learning as a process with a storage buffer (working memory) that has a limited storage capacity C (Eq 2). The limited capacity means that the buffer only stores elements when the condition Q of sufficient storage space is met. For all conditions where Q ≥ 0, the elements (e) are held in serial order:

$$Q=C-\sum_{i=1}^{n}e_{i}$$ (2)

**Fig 1:**
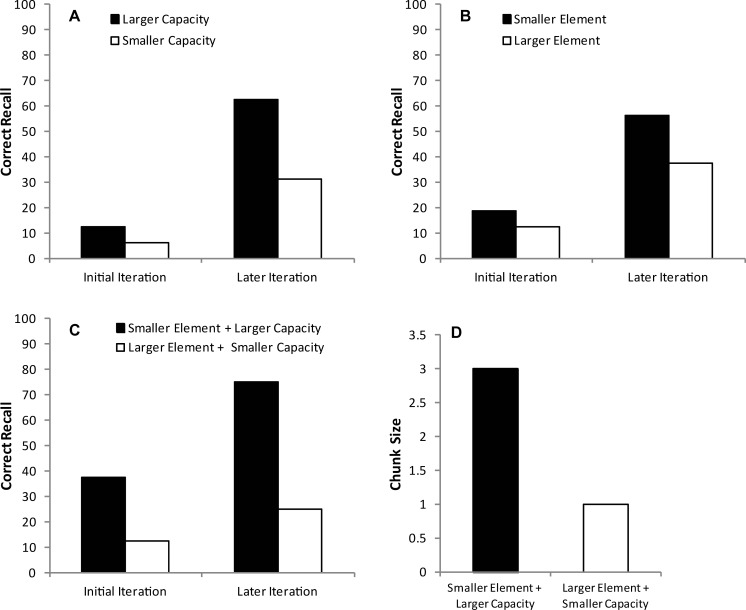
Recall performance when iteratively using Eqs 1 and 2 to model storage of a sequence of movements in the correct order: (A) A smaller working memory capacity will decrease recall on any iteration and lengthen the time taken to learn the whole sequence; (B) A larger element size has a similar effect; (C) The combination of smaller elements and larger capacity will increase recall, whereas larger elements and smaller capacity will decrease recall; (D) A smaller capacity and/or larger element size will decrease the ‘chunk size’–the number of items within a unit written to the longer term storage system.

The buffer can then write the stored element sequence to a longer-term memory store (as a ‘chunk’), and the process repeats iteratively until the whole sequence has been learned. Eq 1 shows that the difficulty associated with any element (measured in terms of information) is directly related to individual skill level and task difficulty, in any activity that involves movement. Thus, increasing the task difficulty increases the informational load of each element and thereby decreases the number of elements that can be stored in any iteration of the learning process. In combination, Eqs 1 and 2 can be used to model sequence learning under circumstances of increased task difficulty or reduced working memory capacity. In the discussion we show how this can be implemented as a model-free reinforcement learning algorithm (though our findings have implications for current computational approaches to modelling learning which typically lack a ‘working memory’ component). Fig 1 shows the qualitative predictions made by the model as a function of storage capacity and element informational content (i.e. difficulty).

While this model is obviously a gross simplification of the learning process, it does capture known features of the cognitive and perceptual-motor system. It also makes clear predictions. First, smaller working memory capacity (or larger element size) will decrease recall on any single iteration and lengthen the time taken to learn the whole sequence (Fig 1A and 1B, respectively). Combinations of smaller elements and larger capacity will also improve recall, whereas larger elements and smaller capacity will impair recall (Fig 1C). Secondly, smaller capacity (or larger element informational load) will decrease the number of items within the ‘chunks’ written to longer-term storage (Fig 1D). Third, an individual with worse motor skill (indexed by the constants in Eq 1) will show slower learning. Finally, increased task difficulty (as defined in Eq 1) will result in a reduced number of elements being learned per iteration (resulting in a longer learning time constant, i.e. more iterations to achieve the same learning).

**Fig 2:**
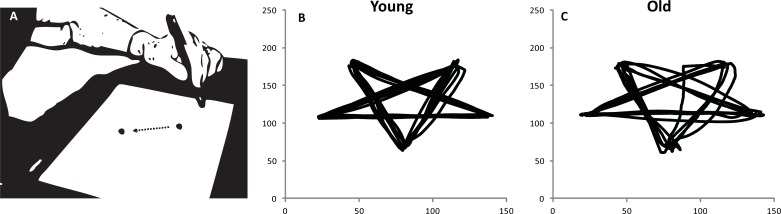
**(A)** Older adult completing an aiming task using a tablet PC and handheld stylus pen (Nb. Not to scale. Arrow for illustrative purposes only). Examples of kinematic traces (speed against time) made in an aiming task by **(B)** a younger adult, and **(C)** an older adult. The spatial paths made by the older participant are much more variable and show more evidence of corrections (which results in quantifiable decreases in smoothness as can be seen in Eq 3 within the text).

We tested this model empirically by measuring the effects of age and motor performance on skill learning in healthy adults. We chose to study aiming movements that were made to discrete target locations because this allowed us to readily define the ‘lower-order’ elements (i.e. the individual movements that underpinned the complete sequence). It is well established that older populations prefer to perform fine motor coordination tasks more slowly [36–39]. A control experiment (Fig 2A; see S1 File for experimental details using methods reported in [54]) confirmed that the quicker movements of younger adults were more direct (Fig 2B) than the longer movement of older adults which were associated with increased error correction compared to a younger group (Fig 2C). To test the relationship between motor performance and learning the first experiment used a novel sequence learning task, suitable for both younger and older adults. The task consisted of training trials that prompted participants to move a mouse cursor to one of eight targets on a screen in a sequence of aiming movements. Following each training trial, learning was assessed by asking participants to attempt to recall the sequence without prompts or feedback. The second experiment employed this learning task in a new set of younger and older participants, with participants using both the preferred (right) or non-preferred (left) hand. Because motor performance is usually worse with the non-preferred hand, our model predicted that this would impact negatively on sequence learning for both age groups. The final experiment compared learning under two levels of motor difficulty using only the preferred hand (to avoid possible confounds linked with hemispheric specialisation): a computer mouse was either used in a standard fashion or oriented sideways on an unfamiliar vertical surface–where reduced motor performance was expected.

## Experiment 1

### Method

#### Participants

Twenty healthy individuals with no previous history of ophthalmological or neurological problems formed an opportunistic sample. All participants were right-handed as indexed by the Edinburgh Handedness Inventory (EHI) [55], with an average score of 96.5 (*SD* = 9.88) out of the maximum 100 (Nb. Scores of 40+ indicate right-handedness). Participants were split into two age groups. The ‘Younger’ group (6 females, 4 males) were aged between 18 and 40 years (mean age = 24.9, *SD* = 7.45) and the ‘Older’ group (6 females, 4 males) were aged between 60 to 75 years (mean age = 69.60, *SD* = 4.12). All participants gave their written informed consent, and the experiment complied with ethical guidelines approved by the University of Leeds ethical committee, in accordance with the Declaration of Helsinki.

#### Procedure and apparatus

**Fig 3:**
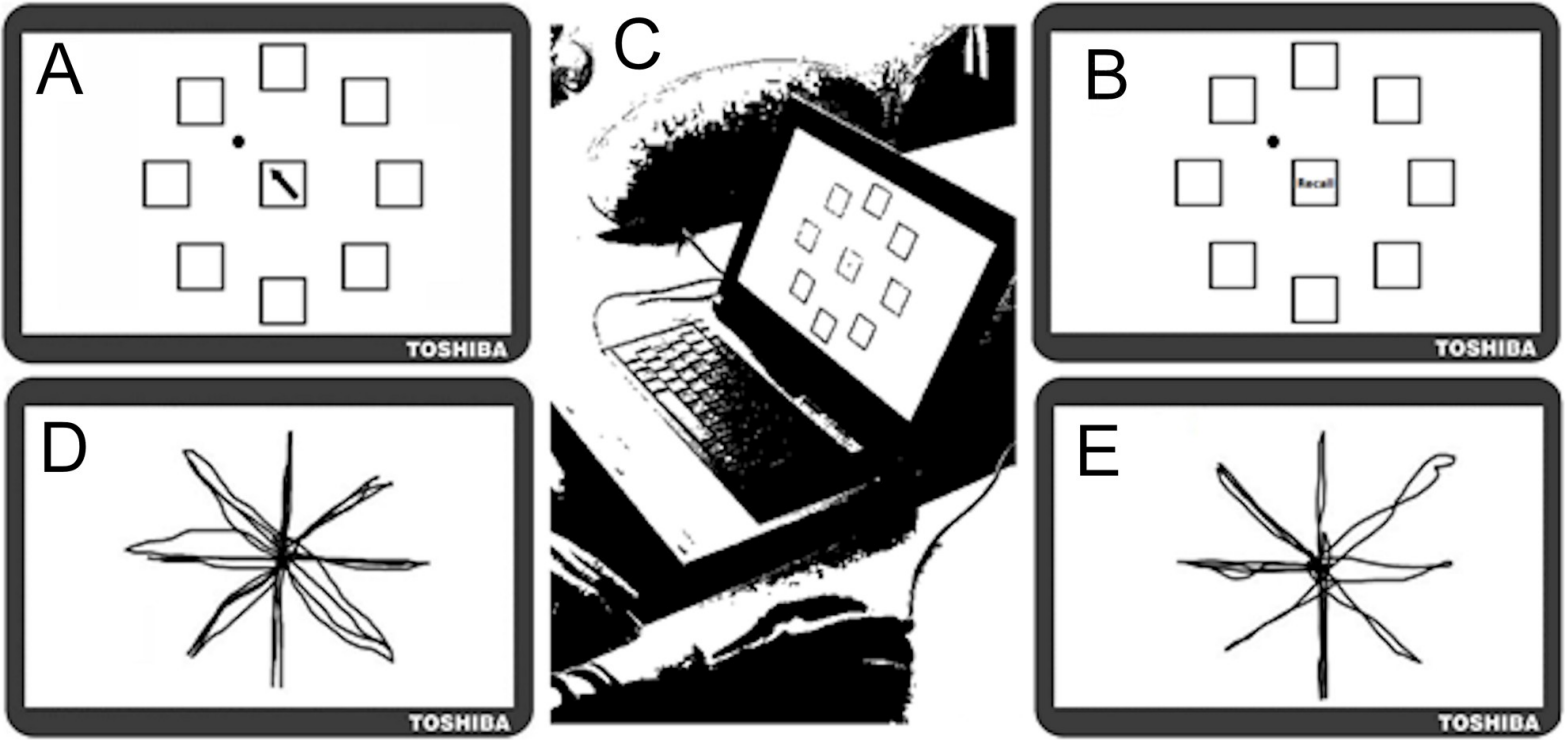
Screen shots of the learning task as it appeared to participants in Experiments 1 and 2 (Nb. not to scale). (A) Training trial whereby participants moved the dot into the box corresponding to the direction indicated by an arrow in the central box (e.g. top left in the example pictured). (B) Test trial in which participants recalled the pattern of movements previously displayed in the training trial. (C) Older adult completing the learning task using a standard computer mouse. Example traces of one participant’s movements during (D) a training trial and (E) a test trial.

A motor learning task was created using ‘KineLab’ [54]. Participants used a tablet PC and standard computer mouse to learn a sequence of movements each made between the centre and one of eight target locations on the screen (see Fig 3C). The task consisted of a series of ‘training’ and ‘test’ trials that alternated to allow 14 opportunities each for participants to practice and then reproduce the sequence (i.e. training trial, test trial x 14 repetitions = 28 trials in total). The same sequence was used across all the participants to ensure that there were no confounding effects created by sequences of differing difficulty. Fig 3A shows the screen as it appeared to participants in the training trial, where there was one central white box (height = 25mm; width = 25mm), encircled by eight identical target location boxes. In the training trials, a black arrow appeared in the central box as a cue for participants to move the circular cursor to the target location adjacent to the direction of the arrowhead (e.g. the correct response would be to move the dot to the top left box for the example given in Fig 3A). After each individual move to a target location, participants returned to the centre at their own pace, at which point the next arrow in the sequence would appear (no mouse clicks were required). There were 30 moves per sequence to learn, which followed an irregular pattern (see example traces from a participant completing the training trial in Fig 3D). All participants received the same training sequence, but the timing of the training was driven by the preferred movement speed of the participant. After each training trial, participants were required to attempt to reproduce the sequence of moves by moving the cursor back-and-forth between the central box and target locations as quickly and as accurately as possible. No feedback was given to participants about whether these movements were correct. Recall trials continued until participants no longer wanted to make movements (either they felt they had completed the sequence or no longer knew where to move). Participants were not instructed when to stop–they could either keep on guessing or stop when they felt unsure about the sequence. Trials were terminated by the participant when they returned and stayed over the centre square for more than 4s. Examples of a training and test trials are shown in Fig 3D and Fig 3E respectively. To ensure participants’ complete understanding of the task, standardised instructions were presented in a series of slides, which included screen shots of the two trial types (similar to those pictured in Fig 3A and 3B). Participants were twice given practice of training and test trials, which featured a 16-move sequence different to that used in the experimental task.

#### Analyses

The following outcome measures were calculated to examine speed and accuracy of recall (i.e. motor learning) in the test trials, and level of motor performance in the training trials.

- *Recall during test trial measures*: Number of moves recalled in the correct sequential order (Correctly Recalled; CR), with a maximum score of 30 (points were not deducted for incorrect moves but in the majority of cases the first error in the sequence marked the end of successful recall); Recall Movement Time (MT_r_) was the mean time (s) taken to move the mouse from the centre to a target box when recalling the sequence (i.e. a measure of recall speed).
- *Training trial measures*: Path Length (PL) indicated the length of the path (mm) taken by participants throughout an entire training trial, thus providing a marker of movement accuracy (i.e. straight paths will be shorter) with longer paths suggesting the presence of error corrections; Training Movement Time (MT_t_) was the time (s) taken to complete a training trial from start to finish.

For the analyses of data from the test trials, mean values for CR and MT_r_ across the first five trials (F5) and last five trials (L5) were calculated. These data were input into two separate mixed-model ANOVAs to compare speed and accuracy of sequence recall between the beginning and end trial blocks (i.e. to identify progression of learning from the first to second half of the task), and between the older and younger age groups. For the training trials, mean values for PL and MT_t_ across the L5 trials were used as a baseline measure of motor performance.

### Results

#### Recall during test trials

**Fig 4:**
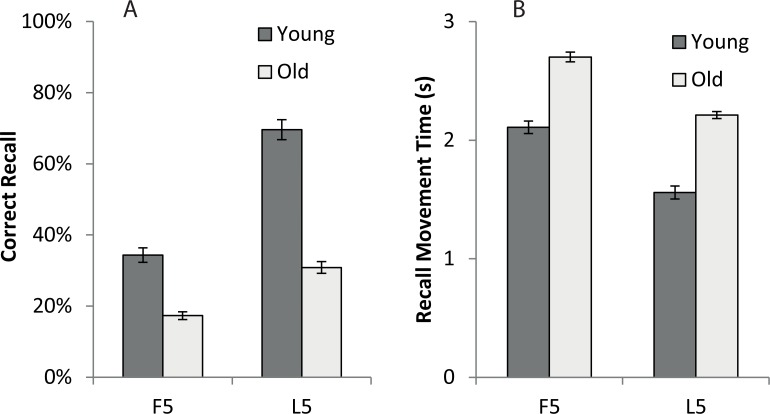
Measurements of motor learning recorded in the test trials of Experiment 1 for younger (dark grey bars) and older (light grey bars) groups, averaged across the first five trials (F5) and last five trials (L5). **(A)** Proportion (%) of movements recalled in the correct sequential order at test (CR) **(B)** Mean time taken between moves during free recall (MT_r_). Bars = Standard Error of the Mean.

The ANOVA for number of moves recalled in the correct sequential order (CR) revealed a significant effect of age (*F* (1, 18) = 15.70, *p* < 0.05, *η*^*2*^_*p*_ = .47), whereby the Younger adults learned a greater number of moves than the Older adults (see Fig 4A). A main effect of trial block (*F* (1, 18) = 50.08, *p* < 0.001, *η*^*2*^_*p*_ = .74) shows that all participants had learned a greater number of moves by the end of the task (mean CR for L5 = 15 items or 50% of the sequence) compared to the first half (mean CR for F5 = 8 items or 27% of the sequence). The interaction between age and trial block (*F* (1, 18) = 9.93, *p* < 0.01, *η*^*2*^_*p*_ = .36), suggests that the Younger group learned disproportionally more than the Older group by the end of all the training.

We also measured speed of recall for correct responses during the task, hence Fig 4B shows the mean Recall Movement Time (MT_r_) for older and younger participants on the F5 and L5 blocks of the test trials. Analyses of the MT_r_ data showed shorter duration movements in the L5 trials compared to the F5 trials (F (1, 18) = 17.06, p < 0.05, η^2^p = .49; mean MT_r_ for the F5 = 2.40s, compared to L5 = 1.89s), and the younger adult group had shorter movement durations than the older adults (F (1, 18) = 11.46, p < 0.05, η^2^p = .39; mean MT_r_ for Old = 2.46s, compared to Young = 1.83s). There was no age ρ trial block interaction (*F* (1, 18) = .06, *p* > 0.05).

#### Training trials

The younger adults demonstrated superior motor performance in the training trials, whereby Training Movement Time (MT_t_) was significantly shorter in the Younger group (*t* (18) = 2.54, *p* < 0.05). There was, however, no reliable age difference in accuracy of aiming movements, as indicated by PL (*t* (18) = 1.25, *p* > 0.05), presumably because the Older adults moved at a slower pace, thus allowing them to maintain comparable accuracy to the Younger adults (i.e. through speed-accuracy trade-offs [36]).

### Discussion

**Fig 5:**
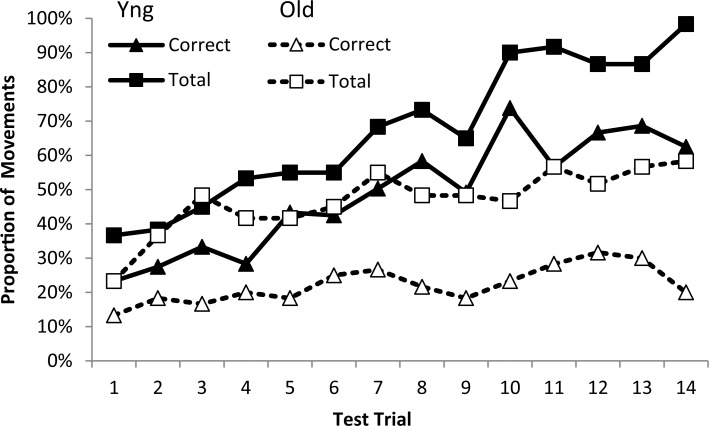
The number of moves recalled during each of 14 test trials by the older (open symbols, dashed lines) or younger (filled symbols, solid lines) groups as a proportion of the total moves in the sequence (proportion of movements). Square symbols show the Total number of moves made, and Triangular symbols show the Correct number of moves made.

The results of Experiment 1 show that the selected task provided a useful measure of movement sequence learning in Younger and Older adults. All participants showed evidence of learning the movement sequence over the set of training trials. The task was neither too difficult (i.e. too little learning), nor too easy (i.e. the sequence learned too quickly), hence it provides a useful metric of learning ability. During test trials participants were free to keep producing movements (including incorrect movements) since there was no explicit feedback for when they started getting the sequence wrong. It was, therefore, possible that younger and older age groups adopted different strategies during recall (it has sometimes been observed that older groups adopt more conservative strategies). Fig 5 shows how many movements were produced relative to the total number of moves in the sequence (as a proportion) for each test trial. It can be seen that both groups adopted similar strategies, making a number of incorrect moves (producing many more Total moves than Correct moves). Initially both age groups produce similar numbers of moves, but by the end of the test trials the Younger group were producing approximately double the number of moves as the Older group. Note that the Older group produce a similar number of Total moves as the number of Correct moves produced by the Younger group, suggesting that there wasn’t a simple physical limitation preventing the production of that number of moves.

This experiment also reinforces previous reports of reduced learning in the Older adults [7]. There are a few possible reasons why the older adults may have shown a reduced ability to learn the sequence. One plausible reason is that older adults have poorer cognitive capabilities (it has been reliably demonstrated that WM declines with age [14, 15]). However we were predominantly interested in whether there might be a relationship between the reduced motor performance of the Older participants and their reduced learning. In other words, we wanted to see whether the model shown in Eq 2 might capture an important feature of sequence learning (the interaction between cognitive and motor constraints). Between-group studies cannot address this question satisfactorily because it is often difficult to disentangle the influence of cognitive differences on learning rates. The next experiment therefore varied the motor performance of Younger and Older adults, *within* individuals, to examine whether sequence learning would be affected.

## Experiment 2

The results of the first experiment are consistent with the hypothesis that there is a relationship between motoric performance level and sequence learning–older participants were found not only to recall fewer moves (than the younger adults) at test, but also moved more slowly during the training trials. Two possible explanations are: (i) that encoding a movement sequence into memory has an influence over the speed of movement (i.e. learning alters motor performance, in this case movement duration), or (ii) that less skilled movements have a causal role in impairing motor sequence learning (i.e. movement performance level affects learning). To distinguish between these explanations a second experiment was conducted on a new set of Older and Younger participants, this time measuring learning when using both the preferred and non-preferred hands. Explanation (i) would predict impaired recall in the Older adults compared to Younger, but no differences between which hand was used to perform the task. Explanation (ii) would predict impaired recall in the Older adults, but also for both age-groups when using the non-preferred hand (i.e. superior motor performance is usually expected in the preferred hand when completing basic motor coordination tasks; see Raw et al. [36]). The same motor learning task was used as in Experiment 2, but because testing needed to be carried out with each hand, the number of movements to be learnt was reduced to keep overall experiment testing time equivalent, to avoid participant fatigue.

### Method

#### Participants

A new group of thirty-seven right-handed healthy individuals with no history of ophthalmological or neurological problems was selected from an opportunistic sample (mean EHI score = 87.40, *SD* = 15.20). We had planned to test forty participants but three did not attend their scheduled testing session. The ‘Younger’ group consisted of 18 participants (11 female, 7 males) aged between 20 and 25 years (mean age = 20.83, *SD* = 1.12), and 19 participants (14 female, 5 males) aged between 61 and 80 years (mean age = 70.79, *SD* = 6.09) formed the ‘Older’ group. The Addenbrooke's Cognitive Examination Revised (ACE-R) [56] was administered to older participants as a measure of basic cognitive ability and the mean score indicated no cognitive deficit at 91.53 out of 100 (*SD* = 5.54). The University of Leeds ethics and research committee approved this study and all participants gave written, informed consent in accordance with the Declaration of Helsinki.

#### Procedure and apparatus

KineLab [54] was used to create two new versions of the task deployed in Experiment 1, each with a different 16-move sequence. Participants completed version one of the task using their preferred (right) hand and version two with their non-preferred (left) hand. The order of which hand/version was used first was counterbalanced across participants (though counter-balancing was incomplete as three participants did not attend). Instructions were the same as for Experiment 1 and participants were given two opportunities to practice the training and test trials, (which included a different 16-move sequence to those used in the experimental tasks). The same hand was used to complete the training and test trials. Each task had 10 training and test trials, resulting in a total of 20 trials per task.

#### Analyses

Outcome measures were identical to those used in Experiment 1 (CR, MT_r_, PL and MT_t_). For the test trial analyses, mean scores across the L5 trials were calculated and two separate mixed-model ANOVAs applied in order to examine age and hand differences in motor learning (CR and MT_r_). Two further ANOVAs were carried out to identify the effects of hand and age on motor performance during the L5 trials of training (PL and MT_t_).

### Results

#### Recall during test trials

**Fig 6:**
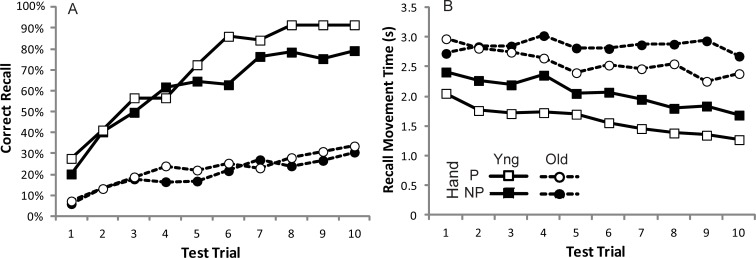
Measurements of motor learning recorded in Experiment 3 for the preferred (right) hand (empty symbols) and non-preferred (left) hand (filled symbols) in the older (dashed line and circles) and younger (solid line and squares) groups for each of the 10 test trials. **(A)** Mean number of moves recalled in the correct sequential order (CR). **(B)** Mean time taken between moves during free recall (MT_r_).

**Fig 7:**
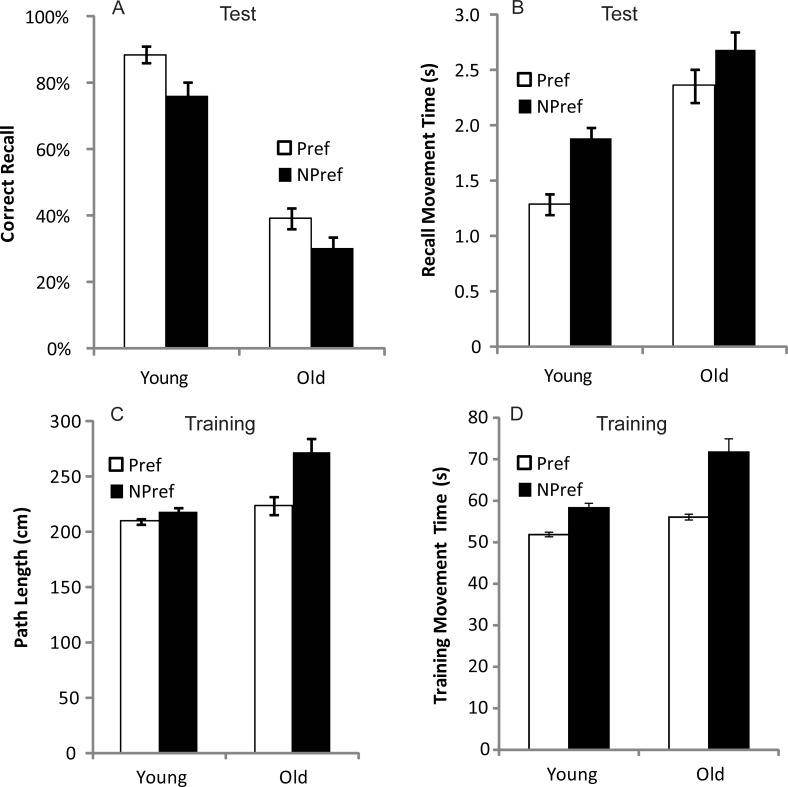
Measurements of motor learning and performance for the preferred (right) hand (white bars) and non-preferred (left) hand (black bars) for older and younger participants averaged across the last five (L5) test trials **(A,B)** and training trials **(C,D)** in Experiment 2. **(A)** Proportion (%) of movements recalled in the correct sequential order at test (CR) **(B)** Mean time taken between moves during free recall (MT_r_). **(C)** Length of entire path taken throughout a training trial (PL). **(D)** Time taken to complete a training trial from start to finish (MT_t_). Bars = Standard Error of the Mean.

Experiment 1 showed that participants became more accurate at recalling the serial order as the trials progressed (Fig 4A and 4B). In Experiment 2, a similar improvement in serial order recall was apparent (Fig 6) but particularly for the Younger adults, and the preferred hand condition. To formally analyse these differences, data from the L5 trials were examined (Fig 7). The ANOVA for CR identified a main effect of age group (*F* (1, 35) = 135.5, *p* < 0.001, *η*^*2*^_*p*_ = .79), a main effect of hand (*F* (1, 35) = 9.13, *p* < 0.01, *η*^*2*^_*p*_ = .21) and a hand ρ age group interaction (*F* (1, 35) = 4.73, *p* < 0.05, *η*^*2*^_*p*_ = .12). Planned comparisons showed that the main effect of hand was driven by a strong effect in the Younger group (F (1, 17) = 10.64, p < 0.01, *η*^*2*^_*p*_ = .39) but no significant effect in the Older adults (F (1, 18) = .47, p = .5, *η*^*2*^_*p*_ = .026). This indicates that the Younger adults recalled a greater number of moves in the correct sequential order than the Older adults, and more moves were recalled by the preferred hand than the non-preferred hand in the Younger adults (see Fig 6A). The hand difference in the Younger group amounts to 2 items fewer recalled by the non-preferred hand. As a proportion of the overall items remembered this is ~12.5% drop off in performance (mean performance for preferred hand over last 5 trials was 14 items). If a similar scale change between preferred and non-preferred hands was to be expected for the older adults then we would predict 0.5 fewer items to be recalled when using the left hand (a 12.5% drop off from the 4.5 items recalled with the preferred hand). It is therefore not surprising that we were unable to measure a hand difference in correct recall for the Older group, given the low level of recall even with the preferred hand. The ANOVA for MT_r_ also revealed a significant main effect of age group (*F* (1, 35) = 34.74, *p* < 0.001, *η*^*2*^_*p*_ = .50) and hand used (*F* (1, 35) = 37.73, *p* < 0.001, *η*^*2*^_*p*_ = .52) but there was no interaction (*F* (1, 35) = .17, *p* > 0.05). Hence younger participants showed decreased movement duration (faster movements) compared to the older group, and the preferred hand was quicker than the non-preferred hand (see Fig 6B).

#### Training trials

It is possible that recall accuracy in test trials could be explained by movement performance during training. To determine whether movement patterns were similar during test and training an ANOVA was run on PL and MT_t_ measures. The PL analysis found main effects of age group (F (1, 35) = 19.72, p < 0.001, *η*^*2*^_*p*_ = .36) and hand condition (*F* (1, 35) = 12.68, *p* < 0.001, *η*^*2*^_*p*_ = .27) as well as an age ρ hand interaction (*F* (1, 35) = 6.12, *p* < 0.05, *η*^*2*^_*p*_ = .15), with the PL difference between the hands being greater in the Older group (see Fig 7C). Similarly, the ANOVA for MT_t_ also revealed effects of age group (*F* (1, 35) = 20.50, *p* < 0.001, *η*^*2*^_*p*_ = .37) and hand (*F* (1, 35) = 46.58, *p* < 0.001, *η*^*2*^_*p*_ = .57) with a significant hand ρ age interaction (*F* (1, 35) = 8.03, *p* < 0.01, *η*^*2*^_*p*_ = .19). Whilst the general patterns were similar between test and training the increased manual asymmetries for the Older group (i.e. a greater difference in movement duration between the preferred and non-preferred hand in the Older adults; see Fig 7D) was not what was observed at test.

#### Chunk length as a function of age and hand

To determine whether there were age differences in the encoding strategies used by participants, a measure of chunking was calculated. We took the average increase in the number of moves recalled in the correct sequential order for each test trial (see Table 1). It is apparent that older adults did not encode chunks of multiple movements on each trial, and instead they generally only increased the number of moves recalled from one trial to the next by a single item. In contrast, the younger adults seem to have stored the motor sequence in chunks of three or four items, certainly over the first three trials (where most of the required moves were stored). Interestingly the chunk size appears larger for the preferred hand in the Younger group, suggesting that motor performance during training can perhaps interact with strategic encoding.

**Table 1:** The average number of additional items recalled in each test trial (over and above those recalled in the previous trial) for older and younger participants when using the preferred (right) and non-preferred (left) hand to complete the sequence learning task in Experiment 3.

		1	2	3	4	5	6	7	8	9	10
**Younger**	Left	3	3.5	2	2	2	0	1	0	0	0
	Right	4.5	3	3	0.5	1.5	2	0	0	0	0
**Older**	Left	0	1	1	0	1	0	1	0	0	1
	Right	1	1	1	1	0	0	0	1	1	1

### Discussion

The second experiment confirmed the results of experiment 1, whereby the Older adults showed reduced sequence learning relative to the Younger adults. It is clearly the case that there are often cognitive differences between younger and older adults [14, 15], and these cognitive differences could explain age discrepancies in an individual’s ability to learn a complex sequence of movements. However, we hypothesised that the reduced baseline level of motor performance typically observed in older adults (i.e. slower, less accurate movements) might also be a contributing factor. This hypothesis was tested in Experiment 2 by asking participants to use both their preferred and non-preferred hand to complete the sequence learning task. The underlying cognitive capabilities of an individual should remain constant regardless of which hand is used to undertake the task, hence differences in learning between the hands would support the prediction (i.e. that reduced motor performance, as typical when using the non-preferred hand, affects motor learning). The data showed that Younger participants learned more of the sequence, and recalled it at a faster pace, when using their preferred hand. These results support the idea that reduced motor performance will impact on complex sequence learning over and beyond any difficulties caused by cognitive decline.

It is notable that age and hand significantly interacted in different directions for the training and test trials. At test, the hand used had an impact on recall in the Younger group, especially during the latter half of the trials (Fig 6). In later trials the Younger adults were producing many more movements, suggesting that this effect may have been cumulative: the more movements that had to be stored and subsequently implemented from memory using the non-preferred hand, the greater the impact of relative motor inefficiency. In contrast, the Older group were only able to produce slightly longer sequences with each new trial (due to limited chunking), thus reducing the opportunity for hand preference to impact on performance. When participants were trying to learn the whole motor sequence in the training trials, however, the effects of reduced working memory capacity and/or processing speed were compounded by reduced motor performance in the non-preferred hand, thus leading to larger effects of hand in the Older group than the Younger group during this phase.

The results of the first two experiments are consistent with the model presented in the introduction. Eq (1) shows that task duration is a function of the informational demand associated with the movement, so further and smaller targets have higher demands and thus result in longer movement times. Eq (1) also shows that the duration of the task is a function of the individual and the task difficulty. Older adults find the given motor task more difficult than their younger counterparts and this can be interpreted as the task having higher informational demands for the older adults. This interpretation is consistent with the presence of errors within the movements made by the older adults–errors that needed to be corrected. The correction of errors requires the allocation of attentional resources and this can explain why these movements use more bandwidth. Eq (2) shows that increasing the information load of individual elements will decrease the number of elements that can be stored in working memory and thus decrease the rate of serial order learning. This suggests that older adults are being affected by both a reduced working memory capacity and the higher information demands created by their reduced motor capabilities.

## Experiment 3

The findings of Experiment 2 suggest that there is a relationship between an individual’s baseline level of motor performance and their ability to learn a novel sequence. When Older and Younger participants used their non-preferred hand to complete the task, we found reduced quality of movements (i.e. longer trajectories and slower movements, during training), and poorer learning (i.e. moves recalled at a slower pace at test, and for the young at least fewer items recalled). This suggests that reduced motor performance can directly inhibit motor sequence learning, over and above the influences of cognitive capacity. An alternative explanation for our results, however, is that learning may be influenced at a neurological level by hemispheric specialisation. According to hemispheric specialisation theory [57], the left side of the brain is predominant in analytical skills such as tasks that involve breaking down problems into parts, reasoning, and logical thinking–the hemisphere said to possess "sequential, analytic, time-dependent mechanisms" [58]. In contrast, the right hemisphere is said to specialise in subjective functions such as intuition, creativity and emotion [59–60]. It might therefore be argued that because participants in Experiment 2 were right-handed, and the contralateral side of the brain that controls right-side movement specialises in skills that are vital for motor sequence learning, participants could have been at an advantage when completing the task with their preferred hand. There are hence two alternative plausible explanations for the finding of reduced motor sequence learning in the non-preferred hand; (i) participants were able to learn more of the sequence when using the hand that is controlled by the left hemisphere because it specialises in the processes involved in motor sequence learning; or (ii) a poor baseline level of motor performance inhibits motor sequence learning.

**Fig 8:**
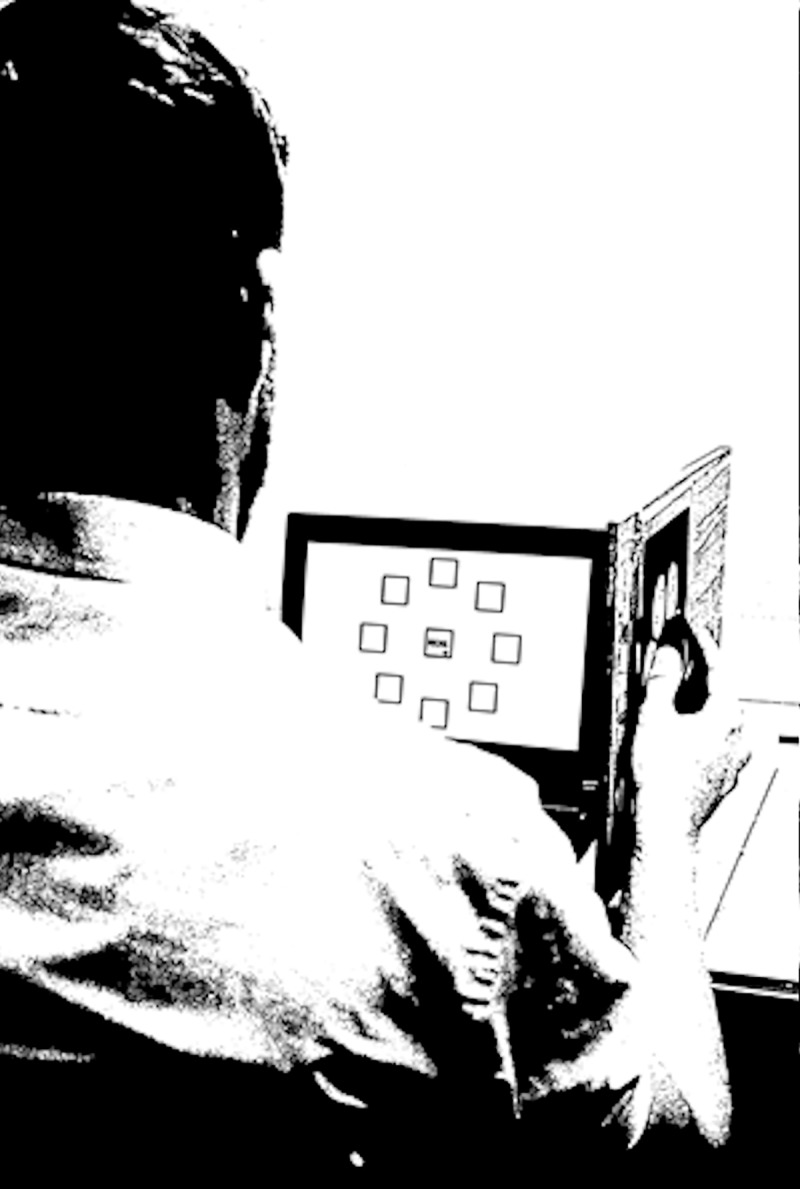
Young adult completing the learning task in Experiment 3, using the mouse in the Sideways (S) orientation.

To tease apart these hypotheses we ran a final experiment using the same sequence learning task, but with two conditions that varied the motor demands placed on the preferred hand. Specifically, participants used their preferred hand to complete the task once when controlling a PC mouse in its regular position flat down on the table (as in Experiments 1 and 2) and then again using the mouse rotated sideways and placed against an inverted T-shaped stand (see Fig 8). The task of using the mouse in a sideways orientation is reminiscent of when an individual is required to carry out a familiar task (e.g. writing) with the non-preferred hand–the action is possible, but less skilled.

### Method

#### Participants

A new opportunistic sample of 15 healthy Young adults (7 female, 8 males) aged between 21 and 40 years (mean age = 29.13, *SD* = 5.64), were recruited. All participants were right-handed (mean EHI score = 87.33, *SD* = 13.87), and reported no history of ophthalmological or neurological problems. The University of Leeds ethics and research committee approved this study and participants gave written, informed consent in accordance with the Declaration of Helsinki.

#### Procedure and apparatus

The sequence learning task from Experiment 2 (created in ‘KineLab’ [54]) was administered under two blocked conditions; (i) participants used their preferred hand to learn the 16-move sequence with a PC mouse in its Regular (R) orientation, flat down on the table (see Fig 4C); (ii): participants used their preferred hand to learn a new 16-move sequence with the PC mouse rotated Sideways (S), and placed up against an inverted-t-shaped stand (Fig 8). The stand was made of wood (width from left to right of base = 620mm; height = 340mm), with an off-centre vertical partition (width = 340mm; height = 310mm). The tablet PC (same as Experiments 1–3) was placed on the left side of the partition, and a mouse mat (width = 230mm; height = 200mm) was secured vertically to the surface of the opposite side. Participants were verbally reminded that when using the mouse in the S orientation, they would need to adapt their movements to account for the change in orientation (i.e. a forward-back motion to produce up-down movements; an up-down motion to make left-right movements). The order in which participants completed the S and R conditions was counterbalanced. At the start of the experiment, participants practiced using the mouse in both orientations, completing one training trial (i.e. whereby participants were shown the 16 targets to move to by centrally placed directional arrows) and one test trial (i.e. free recall of the sequence practiced in the training trial) with the mouse in the R or S orientations (NB. the practice sequence used was different to the sequences used in the experimental tasks). The experimental task comprised 10 training and test trials, hence 20 trials in total for each mouse orientation condition. The same mouse orientation was used to complete the training and test trials.

#### Analyses

Outcome measures were identical to those used in Experiments 1 and 2 (CR, MT_r_ for the recall at test analyses; PL and MT_t_ for the training trial analyses). For recall during test trials, mean scores for CR and MT_r_ across the last five (L5) trials were calculated, and a One-Way ANOVA was performed on each recall measure, to examine the effect of mouse orientation (R vs. S) on motor learning. A One-Way ANOVA was also completed to identify the effects of mouse orientation on motor performance during the L5 training trials (one for each training measure: PL and MT_t_).

### Results

#### Recall during test trials

**Fig 9:**
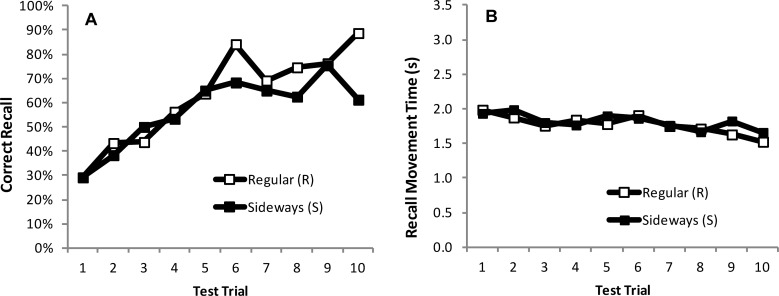
Measurements of motor learning of the preferred (right) hand with the mouse in the Regular (R) position (empty symbols), and the Sideways (S) position (filled symbols) for each of the 10 test trials in Experiment 3. **(A)** Mean number of moves recalled in the correct sequential order (CR). **(B)** Mean time taken between moves during free recall (MT_r_).

As previously observed in Experiments 1 and 2, participants were able to recall more of the motor sequence, and at a faster pace, as the task progressed. It can be seen in Fig 9 that differences in motor learning (indexed by CR) began to materialise between the mouse orientation conditions in the second half of the task. We therefore analysed data from the last five (L5) test trials to formally explore these differences.

**Fig 10:**
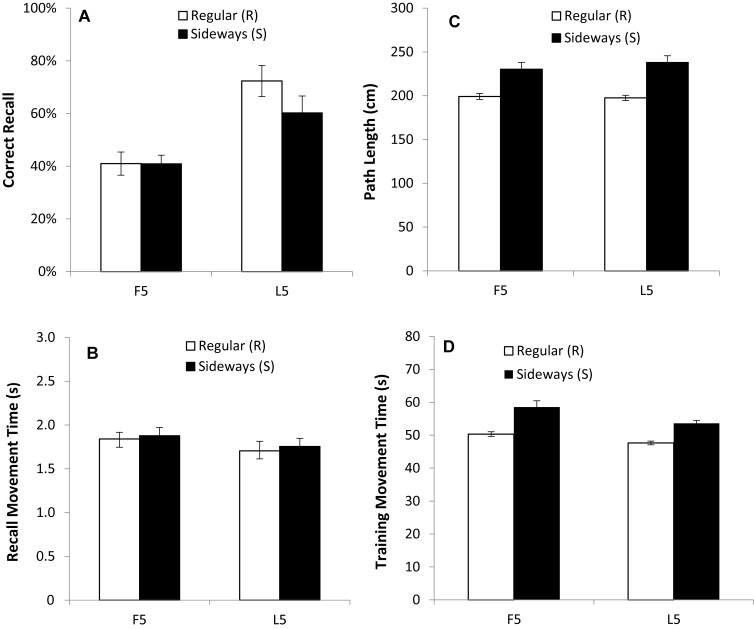
Measurements of motor learning and performance of the preferred (right) hand with the mouse in the Regular (R) position (white bars), and the Sideways (S) position (black bars), averaged across the first five (F5) and last five (L5) test trials **(A, B)** and training trials **(C,D)** in Experiment 3. **(A)** Proportion (%) of movements recalled in the correct sequential order (CR) at test **(B)** Mean time taken between moves during free recall (MT_r_). **(C)** Length of entire path (PL) taken throughout a training trial. **(D)** Time taken to complete a training trial from start to finish (MT_t_). Bars = Standard Error of the Mean.

One-Way ANOVAs of the CR data revealed a main effect of mouse orientation (*F* (1, 14) = 4.99, *p* < 0.05, *η*^*2*^_*p*_ = .26; Fig 10A), whereby participants were able to recall significantly more moves when using the mouse in the R orientation (mean CR = 13 items or 81% of the sequence), compared to the S orientation (mean CR = 11 items or 69% of the sequence). The speed at which participants were able to recall the moves (MT_r_) was similar for the S and R orientation conditions (mean MT_r_ for S = 1.75s; mean MT_r_ for R = 1.70s; Fig 10B), hence there was no main effect of mouse orientation on MT_r_ (*F* (1, 14) = .28, *p* > 0.05; Fig 10B). To ensure that the sideways conditions did indeed cause motoric difficulties we next looked at performance during the Training Trials.

#### Training trials

To confirm a significant reduction in motor performance in the S condition we analysed speed (MT_t_) and accuracy (PL) of movements recorded during the training trials (using separate One-Way ANOVAs). Analyses revealed main effects of mouse orientation for both PL (F (1, 14) = 34.84, p < 0.001, *η*^*2*^_*p*_ = .71; Fig 10C) and MT_t_ (F (1, 14) = 91.32, p < 0.001, *η*^*2*^_*p*_ = .87; Fig 10D) whereby PLs were significantly longer, and movements slower (mean MT_t_, for S = 54s; mean MT_t_, for R = 48s), when the mouse was used in the S orientation. It seems that it is the increased motor difficulty exhibited during training that is having the detrimental effect upon recall performance, because there were only small differences in movement times at recall (MT_r_).

### Discussion

In Experiment 2 we observed reduced sequence learning in the non-preferred hand. This effect may have been due to differences in hemispheric specialisation (i.e. the left hemisphere, which controls the right hand, could have been dominant in the processes vital for sequence learning). Alternatively, this effect could be caused by a reduced baseline level of motor performance (i.e. as typical of the non-preferred hand) that directly inhibits motor sequence learning. Experiment 3 aimed to distinguish between these explanations by controlling task difficulty of just the preferred hand–comparing learning when using the mouse in its Regular (R) orientation and in an unfamiliar sideways (S) orientation (i.e. hemisphere use and individual differences in cognitive capacity remain stable between conditions). The findings of Experiment 3 confirmed our original hypothesis–sequence learning was reduced when participants used the mouse in the S orientation (i.e. as indexed by fewer moves recalled in the correct sequence order at test), and this was coupled with significantly poorer accuracy and decreased speed of movements (i.e. both markers of poor motor performance) during training.

#### General discussion

The Cognition-Action Interaction Theory (CAIT) suggests that the acquisition of many skills will involve a synergistic interplay between the cognitive and motor systems. We hypothesised that increasing the difficulty of the motor elements within a complex sequence learning task would decrease the number of elements that could be held in working memory, and thereby reduce the rate of learning. Our hypothesis was based on the observation that increasing motor difficulty increases the informational load. For example, increased difficulty is likely to require more error corrections, with such corrections consuming attentional resources and thus decreasing the system’s available bandwidth. We created a simple model (see Eq 2) that captured the increased informational load associated with task difficulty and the limited capacity of working memory. The model predicts reduced learning with decreased cognitive capacity as well as with reduced motor abilities (both established as being present in older adults). We tested the model by exploring whether increased task difficulty impaired sequence learning.

Experiment 1 revealed reduced movement speed and increased path length (indicating error corrections) in older adults when making similar aiming movements during the training phase of a sequence learning task (i.e. reduced motor performance, as indicated by increased movement duration). Moreover, the older adults showed poorer learning outcomes. Experiment 2 examined whether motor performance directly affected movement sequence learning (as predicted by our model) by having a different group of older and younger participants perform the learning task with their preferred or non-preferred hands. The results confirmed that the older participants learned less of the sequence than the younger participants, and that use of the non-preferred hand reduced learning compared to the preferred hand. At recall, movements with the non-preferred hand were slower in both age groups and the number of correctly recalled items was significantly impaired for the younger adults. This suggests that sequence learning can be influenced by the underlying motor performance as predicted by CAIT. Overall, the present findings support the conjecture that the motor and cognitive systems both play essential but interacting roles in skill learning. This raises the question of whether motor performance could have been influenced by the quality of the memory formed for the movement sequence itself. While the acquisition of accurate memory representations does support subsequent skilful execution of movements, within the context of the current task it appears that motor performance affected the task learning, rather than the reverse. This was most clearly observed in Experiment 3 where slower and less accurate movements during training led to worse recall despite there being no apparent motor performance differences when recalling at test.

Our model (see Eq 2) demonstrates that motor performance will not explain all the group differences that were observed in Experiments 1 and 2. Fig 1A demonstrates the predicted change in performance when working memory capacity is set to a lower value which effectively models the capacity of older adults. These predictions match our findings: for example, motor performance during the training phase of Experiment 2 (MT_t_) was similar for the non-preferred hand of the younger adults and the preferred hand of the older adults (Fig 7D), but there was a 45% reduction in the number of correct movements recalled by the older adults compared to the younger adults with these hands (Fig 7A). Not only was this difference predicted by the model, but the model captured a reduction in chunk size for the older group (because chunk size is limited by working memory capacity as described in Fig 1D). To examine the group differences, chunk sizes used by the older adults and younger adults when recalling the sequence were calculated and are displayed in Table 1. Younger adults used a standard chunk size of 3–5 items (particularly when recalling the first 10 items), which is comparable with previous research [17]. Such chunking during sequence learning improves processing efficiency and is thought to be critical in representing lengthy sequences [18]. In contrast, the older group did not effectively add chunks to their overall representation of the sequence on each trial. Instead, the number of items recalled tended to increase by a single item at a time. This pattern fits with previous research showing age-related impairments in chunking [17, 19] and association-formation [28]. The study by Bo et al. [17] showed impaired learning of sequences in older adults; specifically, visuo-spatial working memory ability was found to indirectly predict learning in older adults via a mediating effect on the size of chunks that could be constructed. It therefore appears that age differences in learning some skills are at least partly the result of reduced working memory capacity constraining the size of chunks that can be built on each trial–but our results show that this effect will be magnified when there are higher motor demands.

The model we present is consistent with reinforcement learning frameworks and can be implemented within such schemes. Q-Learning [61] is a model-free reinforcement learning algorithm, through which an agent learns how to act in an environment. In Q-learning, tasks are modelled as a Markov Decision Process (MDP), which is a tuple: *M* = 〈*S*,*A*,*p*,*r*〉 where S is the set of states, A is the set of actions, and *p*(*s*_*t*+1_,*r*_*t*+1_|*s*_*t*_,*a*_*t*_) is the joint distribution over the next state and reward given the current state and action, and r:S×A×S→R is the reward function. The interaction proceeds in discrete steps: the agent chooses an action based on the current state according to a policy represented by a probability distribution *π*(*a*_*t*_|*s*_*t*_), and receives the perception of the next state *s*_*t*_, and immediate reward *r*_*t*_. The agent’s goal is to maximize the long-term cumulative reward, called the return $G_{t}=\sum_{t=1}^{T}r_{t}$, where *T* is the maximum number of actions in the task. Q-learning is used to learn an estimate of the value function $q_{\pi^{*}}(s_{t},a)=E{|}_{\pi^{*}}[G_{t}]$ from the information provided by the samples of states and rewards—that is, the expected value for taking action a in state *s*_*t*_ when acting according to the optimal policy. The estimate ${\hat{q}}_{\pi^{*}}$ of $q_{\pi^{*}}$ represents the long-term memory of the agent, and is used to determine the policy of the agent during learning. Following learning (i.e. once the estimate of the value function is close to the true optimal value function), the optimal policy can be retrieved simply by choosing the action that maximizes the q-value in every state. An agent is normally naïve with respect to the environment, so starts with all state-action pairs having the same estimated value. Typically, after an action the state-action valuation is immediately updated, but the work reported within this manuscript suggests that the working memory process could be represented at a basic level by adding new information to a fixed-capacity queue. When a new piece of information is generated, a check would be conducted to only add the item to working memory if it would “significantly” change the existing value function. A test of recall would then be a measure of the agent’s attempt to use the current estimate of the optimal value function, ${\hat{q}}_{\pi^{*}}$ to complete the task.

**Fig 11:**
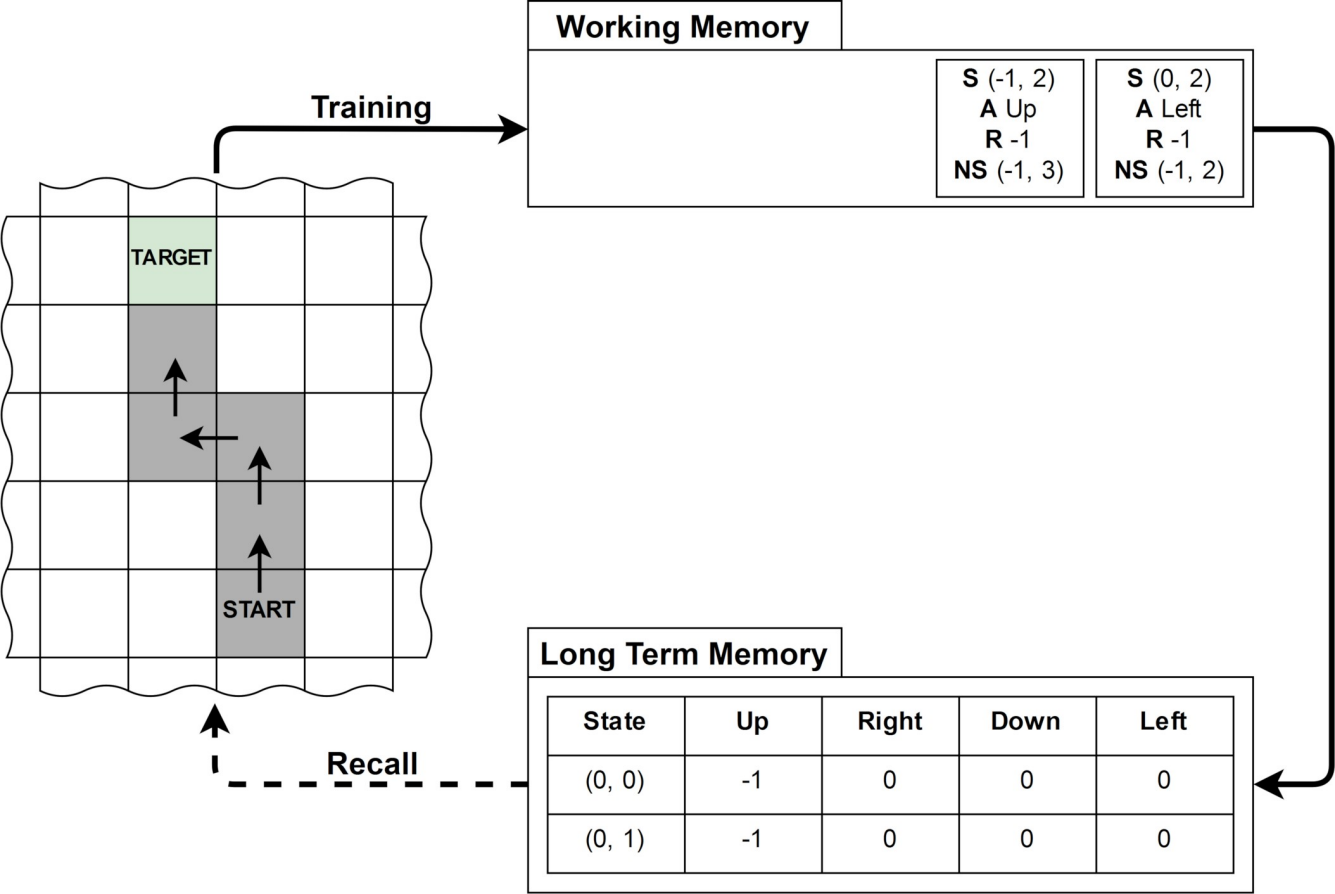
Schematic of the model implementation within a Q-learning framework.

Fig 11 illustrates the postulated process within a simple grid world where the state space is constituted by the cells of the grid, and the actions are to move in one of the four cardinal directions. The environment is deterministic, so each action always leads to the state in the corresponding direction, except for when it would lead the agent outside of the grid in which case the agent stays in its current state. The reward is -1 per action, and an episode ends when the agent reaches a target state, or has executed the maximum number of actions. If we apply this system to the sequence learning task reported in Experiments 1–3, each target in the sequence has its own 2D grid and q-function that needs training. The working memory system is then simply implemented by a queue that rejects new items once it is full and updates long-term memory at the end of an iteration (note this can be implemented in a number of possible ways to suit an application or to capture features like recency effects). Under this system, only early goals in the sequence are initially trained, but once these goals have been ‘learned’ then new information for these goals does not need to be committed to working memory, so the next goals in the sequence can be trained until all goals are learned via an iterative process. In this implementation, task difficulty can be represented by the number of steps required to reach a target (which captures the notion that more difficult tasks require more information). The differences we found between preferred and non-preferred hand use (Experiment 2) can then be explained by differences in the accuracy of the agent’s existing models of the environment.

It should be highlighted that the notion of a synergistic interaction between cognition and action in complex tasks is not equivalent to the hypothesised interference that occurs in dual tasking. Dual tasking can be a useful conceptual framework when considering activities that are readily dichotomised as being either distinctly motor or cognitive in nature. The classic example is maintaining posture whilst undertaking mental arithmetic. In this situation, the goal of the motor task (not falling over) can be argued to be separate from the goal of the cognitive task (answering the maths question), and hence serves as a useful tool for exploring the extent to which these separate processes share neural resources. A problem, however, is that success in numerous human activities (including the task used within the present study) requires a combination of both motor and cognitive elements, and it becomes meaningless to conceptualise the outcome of one system as being separate to the goal of the other system. Thus, our model does not depict the motor system as interfering with the cognitive system, but rather demonstrates that these two systems are mutually dependent when learning a complex skill (and sometimes share neural resources–as explored in some dual-task experiments). It is also interesting to note that the research literature suggests that complex motor behaviours become increasingly ‘cognitive’ (i.e. less reliant on automated motor routines and requiring grater cognitive resource) as adults become older [62].

The ability for procedural memory to inform the necessary sequence of actions to achieve a goal is crucial for many activities of daily living (e.g. tying a shoe-lace), but it also underpins highly skilled and risky activities such as driving or carrying out complex surgical procedures. Such highly practiced abilities, eventually stored as procedural knowledge, must initially be acquired through learning processes that are much more resource-intensive and controlled [63–65], and require construction and temporary storage in working memory. It is this initial learning phase that was examined in the present experimental work. Temporary storage and control processes are likely to become less critical once the initial stages of learning have progressed and procedural memory develops. In line with this, Sakai, Hikosaka & Miyauchi et al. [12] suggested a shift in the importance of certain brain regions during the transition from declarative to procedural memory in visual-motor sequence learning; whereby early learning primarily activates frontal areas, particularly the dorsolateral prefrontal cortex (DLPFC) and presupplementary motor area (pre-SMA), with a shift to parietal areas as sequences become consolidated. The observed age and hand effects in Experiment 2 may reflect the potential roles of the DLPFC and pre-SMA in initial visuo-motor learning.

The findings of our experiments have important implications within applied settings. For example, CANTAB sells “cognitive assessment and data collection software” (Cantab Connect Research Alzheimer’s) that purports to test cognitive function in older adults. The software has a test for paired associate learning which requires participants to touch boxes displayed around the screen. This test clearly has a motor component (the task is not dissimilar to the one reported in our experiments) and is advertised as providing “insight into individuals’ episodic memory abilities”. The present findings show that such tests need to consider the motor abilities of the participants–and these ‘cognitive’ measures absolutely must be interpreted in terms of their interaction with the motor system.

While the present work examined some of the deficits associated with old age, we would argue that the conclusions drawn from our results can also be generalized to other groups that experience motor deficits. One example is children with developmental coordination disorder (DCD), who make up approximately 5% of the population [6]. Children with DCD experience a host of related problems that often become particularly apparent in mainstream education. Movement difficulties could lead to greater demands on working memory within many school learning tasks (e.g. following a set of instructions relating to the placement of objects within the classroom). Working memory itself provides a good predictor of scholastic ability [66] but given the co-morbidity of DCD with other developmental problems there could well be complex interactions between memory and motor deficits (resulting in poorer educational outcomes for these groups of children). We note that numerous classroom activities require a complex interaction between cognitive and motor systems (e.g. handwriting involves producing words which must be mentally manipulated whilst concurrently controlling the forces applied to a handheld stylus). In line with this, it has been found that children with poor working memory have problems in following and implementing instructions within the classroom [67], and that working memory tasks measuring this ability may have an important motoric component [68].

The present experiments show that increased motor demands can impair sequence learning and better motor performance is associated with improved sequence learning. We conclude by highlighting that the interactions between cognition and action may have numerous positive effects that can be exploited within clinical settings. For example, information can be stored within the motor system and thereby decrease the storage and retrieval demands placed on cognition. Recent findings indicate that the motor component of complex movement tasks may have such facilitating effects, with recall being improved when miming an action, for example recalling the digit sequence of a PIN number when the spatial layout of the keypad is present [69]. A key priority for future research should be to determine *how* action and memory interact, to fully understand the performance of skilled actions. This will also help to identify what causes errors in critical tasks, such as driving or surgery, and how to best support individuals who show deficits in learning skilled behaviours.

## Supporting information

S1 File Control experiment establishing impaired motor performance by older adults performing perceptual-motor aiming tasks similar to those used in Experiments 1–3. (DOCX)
