# Supplementary material for: Skill acquisition as a function of age, hand and task difficulty: Interactions between cognition and action
Source: PLoS One. 2019 Feb 7;14(2):e0211706. doi: 10.1371/journal.pone.0211706 (PMC6366788; doi:10.1371/journal.pone.0211706)
Supplement: S1 File — (DOCX) [file pone.0211706.s001.docx]

# S1 File

This control experiment established impaired motor performance by older adults performing perceptual-motor aiming tasks similar to those used in Experiments 1, 2 and 3.

## Method

### Participants

Forty healthy individuals with no previous history of neurological problems formed an opportunistic sample. We ensured that the participants could see the stimuli clearly (and were wearing the appropriate refractive correction) and that they had no history of ophthalmological difficulties. We were therefore confident that the visual stimuli were well above threshold so that visual difficulties per se were not a confounding factor. All participants were right-handed as indexed by the hand participants stated that they used to write. Participants were split into two age groups. The ‘Younger’ group (6 females, 14 males) were aged between 20 and 27 years (mean = 21.95, *SD* = 1.76) and the ‘Older’ group (6 females, 14 males) were aged between 61 to 92 years (mean = 74.5 *SD* = 9.55). All participants gave their written informed consent, and the experiment complied with ethical guidelines approved by the University of Leeds ethical committee, in accordance with the Declaration of Helsinki.

### *Procedure and Apparatus*

An aiming task was created using ‘KineLab’, a kinematic assessment tool that can be used to design visual-spatial tasks and record the x and y co-ordinates of hand movement [54]. Participants used a handheld stylus, which was a similar shape to a ballpoint pen (stylus length = 150mm; nib length =1mm), to complete the task on a tablet PC (screen width = 260mm; screen height = 163mm). All participants completed the aiming task using their preferred (right) hand and instructions appeared on the screen before the task commenced. Participants were asked to move the pen as quickly as possible from one dot (diameter = 10mm) to another as they appeared on the screen (distance between dots = 117mm). The appearance of the dots followed the shape of a pentagram, repeating 10 times throughout the duration of the task (i.e. 5 moves per repetition to complete the pentagram shape giving a total of 50 trials; see Fig 2). Participants needed to have the stylus entirely within the target area for 250ms, for it to be considered a successful trial, with the next target appearing immediately after the previous trial had successfully concluded. We defined the start of a movement as the time point when the participants left the start location on the screen and the end of the movement as the time point when the participants arrived in the target location and stayed there for 250ms (i.e. just moving through the target location would not be taken as the end of the movement). This allowed us to identify the start and end of the movement. We could then mathematically differentiate the position to produce a ‘speed profile’ and obtain measures of the peak speed and the time at which peak speed occurred (see Culmer et al. [54] for details). This allowed us to determine the deceleration time. We quantified smoothness using a normalised jerk index given by:

$Nj= \sqrt{\frac{T^{5}}{2L^{2}}\int_{0}^{T} j\left( t \right)^{2}dt}$ (3)

In this equation, T = Movement Time; L = Path Length; j(t) = δ^3^x/δt^3^, x = Displacement. We normalised jerk with respect to time and distance so that trajectories of different durations and lengths could be compared (the measure is consequently unit less). A maximally smooth 1D trajectory that starts and ends at rest is described by a quarter cycle of a sine wave, which gives a normalised jerk of 7.75. The metric was extended to 2D by finding the resultant tangential velocity of the movement, then differentiating twice to find the resultant jerk. This metric is particularly sensitive to measurement noise because the jerk is obtained by differentiating a position signal three times, thus amplifying high frequency noise components. We therefore implemented a second order low-pass zero-phase Butterworth filter to attenuate high frequency noise from the signal and prevent it from contaminating the normalised jerk measurement.

### Analyses

Mean values across all of the movements made throughout the aiming task for three outcome measures of performance were calculated; (i) *Movement Time (MT)*, the time taken to move the stylus between two dots (i.e. where lower MT equates to higher performance level); (ii) *Deceleration Time (DT),* the time between the point of reaching peak speed and stopping. Smaller values (in sec) indicate less time taken to decrease speed from peak to zero, whereas longer DT’s suggest a greater period of time spent in the phase where online corrections are made: (iii) *Normalised Jerk (NJ),* a measure that captures changes in acceleration, with higher values indicating less smooth and more variable movements (i.e. an index of the number of corrections made within a movement). To examine the effects of age on these measures of aiming performance, three independent Samples t-tests were applied.

## Results

The Movement Time (MT) data showed a significant effect of age on participants’ movement duration, whereby the older adults (mean MT = 1.56s) made slower aiming movements than the younger adults (mean MT = 1.11s; t (38) = 6.75 p < .001). Furthermore, the t-test for Deceleration Time (DT) showed significantly longer DT in the older group (mean DT = 0.41s), compared to the younger (mean DT = 0.33s), suggesting that the older participants spent more time in the error-correcting phase of a movement. In line with this finding, a significant between-group difference in Normalised Jerk (NJ; t (38) = 2.57 p < .05) showed that the traces made by older participants were less smooth (mean NJ = 1504.26) than those of the younger (mean NJ = 165.07), again demonstrating the presence of corrective adjustments in the older group (i.e. corrective adjustments cause less smooth, and somewhat ‘bumpier’ traces). This difference in smoothness can be seen clearly in Fig 2, which shows the spatial paths made by one younger (Fig 2b) and one older (Fig 2c) participant during the aiming task.

## Discussion

These results confirm previous reports of increased movement duration in Older adults (e.g. Raw et al., 2012a; 2012b), in this case, when carrying out a series of aiming movements, with no learning requirement. The results can be interpreted in the context of Equation 1, which shows that duration is a function of the information load associated with movements to further and smaller targets, but is also a function of the individual, and the task. Equation 1 shows that the older adults find movements of a given level of extrinsic difficulty (i.e. the size and distance of the movements was equal for both groups) more challenging than younger adults. This can be interpreted in terms of the information load associated with these movements as a function of age (i.e. the movements have higher information loads for older adults). Further evidence of reduced motor performance in the Older group was also confirmed by the analyses of DT and NJ data – the Older participants spent more time in the error-correcting phase of movement (i.e. increased DTs compared to the Younger adults), and their traces were more variable and less smooth than those of the Younger group (i.e. increased NJ showing the presence of corrective adjustments). The increased movement duration observed in the Older age group (and greater variability in motor performance) supports the prediction that the Older adults may have increased difficulty learning a sequence of aiming movements because of the capacity limits of working memory (independent of age-related cognitive decline).
